# A Study on Deliberate Self-Harm by Poisoning and Associated Risk Factors at Batticaloa Teaching Hospital

**DOI:** 10.7759/cureus.73971

**Published:** 2024-11-19

**Authors:** Suranga Singhapathirana, Pakkiyaretnam Mayurathan

**Affiliations:** 1 Department of Medicine, Batticaloa Teaching Hospital, Batticaloa, LKA; 2 University Medical Unit, Batticaloa Teaching Hospital, Batticaloa, LKA; 3 Department of Clinical Sciences, Faculty of Health Care Sciences, Eastern University Sri Lanka, Batticaloa, LKA

**Keywords:** deliberate self-poisoning, poisoning, public health interventions, socioeconomic factors, suicide

## Abstract

Background: Deliberate self-harm (DSH) by poisoning is a significant public health concern worldwide. This study aimed to analyze the demographic, socioeconomic, and psychological factors contributing to self-harm by poisoning in patients admitted to a tertiary care hospital in Sri Lanka.

Methods: A cross-sectional study was conducted on 101 patients admitted to the hospital for poisoning-related self-harm. Data was collected through a structured questionnaire. Variables such as age, gender, educational background, employment status, income levels, substance abuse history, type of poisons used, sources of knowledge about self-harm methods, immediate stressors, and other few possible factors were analyzed.

Results: The majority of patients, 55 (54.5%), were between the ages of 18 and 29. Fifty-five women (54.5%) slightly outnumbered 46 men (45.5%). More than half of the included patients had an educational background of up to ordinary level exam, while 58 were unemployed (57.4%), including dependents. Lower income levels were associated with higher rates of self-harm. Substance abuse, particularly alcohol, was prevalent in 34 (33.7%) of cases. The most commonly used poison was yellow oleander, reported in 38 (37.6%) of cases. The study also found that a majority, 53 (52.5%), learned about self-harm methods through community engagement. Immediate stressors, including arguments with parents, 29 (28.7%), and conflicts with spouses, 19 (18.8%), were significant triggers. Most patients, 76 (75.2%), were admitted to the hospital within six hours of poisoning. Half of the patients, 50 (50%), reported that immediate stressors directly related to the COVID-19 pandemic significantly influenced their decision to self-harm. Regarding patient outcomes, the study found that nearly all patients, 100 (99%), were successfully discharged from the hospital after treatment.

Conclusion: This study provides valuable insights into the factors contributing to DSH by poisoning in a Sri Lankan context. Targeted interventions addressing the identified risk factors, along with broader public health strategies, are essential to reduce the incidence of self-harm.

## Introduction

Deliberate self-harm (DSH) is a significant and growing public health concern globally, characterized by individuals intentionally inflicting harm upon themselves without the intent of suicide [1]. Among these, deliberate self-poisoning is particularly prevalent and poses severe health risks due to the potential for acute toxicity and long-term physical and psychological consequences [2]. Each year, approximately 0.7 million people worldwide die by suicide, with many more attempting it [1]. An estimated 20% of these suicides result from self-poisoning, predominantly in rural agricultural areas of low- and middle-income countries [3]. The Asia-Pacific region has reported a higher annual death rate due to deliberate self-poisoning [2].

In Sri Lanka, deliberate self-poisoning is a significant concern, with over 80% of self-harm cases involving the ingestion of poisons or drug overdoses [3]. This public health challenge is exacerbated by the availability of toxic substances, such as pesticides, and has placed a heavy financial burden on the healthcare system [4]. Despite efforts to reduce suicide rates by restricting access to lethal substances, the rate of suicide attempts and self-poisoning in Sri Lanka remains high at approximately 15 per 100,000, contributing to significant healthcare costs. Sri Lanka's suicide rate continues to exceed the global average of around nine per 100,000, with self-poisoning still a prevalent method [5]. The distribution and choice of poisons vary significantly across different regions within Sri Lanka, influenced by factors such as geography, availability, ease of access, socioeconomic conditions, and cultural and religious beliefs [6].

The Batticaloa district in Sri Lanka, with a predominantly agricultural population, has been particularly affected by self-poisoning incidents. Cultural, economic, and geographic factors influence the types of poisons used in these cases. Previous studies in Sri Lanka have highlighted the prevalence of poisoning by substances such as organophosphates and plant seeds like *Cerbera manghas*, though recent comprehensive research is lacking [7-9].

Given the socioeconomic challenges, including the COVID-19 pandemic and ongoing economic crisis, this study aims to investigate the demographic patterns, risk factors, and outcomes associated with DSH by poisoning at Batticaloa Teaching Hospital. This hospital-based study will help to understand the current state of the issue and provide valuable data for developing prevention strategies and enhancing hospital-based management protocols.

## Materials and methods

This cross-sectional descriptive study was conducted at Batticaloa Teaching Hospital, from January to November 2022, after obtaining approval from the Ethical Review Committee of the Faculty of Health Care Sciences, Eastern University Sri Lanka (approval number: E/2022/52), to analyze the characteristics and outcomes of patients admitted for deliberate self-poisoning. The study population consisted of all individuals aged 14 years and older who met the inclusion criteria of deliberate self-poisoning and provided informed consent to participate. The study excluded cases of accidental or homicidal poisoning, individuals under 14 years of age, and cases related to snake bites, insect bites, or food poisoning to maintain a specific focus on DSH.

Complete enumeration was employed, allowing for the inclusion of every patient fitting the study's criteria over the 11-month period. This comprehensive approach eliminated the need for sampling and ensured that the full spectrum of patients admitted for self-poisoning was represented. Data was collected through a structured questionnaire, administered by an interviewer in both local languages and English. The questionnaire captured detailed clinical, demographic, and psychosocial information, including age, gender, educational background, employment status, income level, previous substance abuse, sources of poisoning knowledge, and the impact of the COVID-19 pandemic.

Patients were divided into six age groups: 14-18, 18-29, 29-39, 39-49, 49-59, and 60 or older. Educational background was categorized into five groups: illiterate, up to primary school, up to ordinary level exam, up to advanced level exam, and tertiary level education. Employment status was classified as student or dependent, unemployed, and employed, while income levels were grouped into six categories based on monthly earnings in Sri Lankan rupees (LKR). Additionally, the study explored the previous substance abuse patterns of patients, including the use of alcohol, smoking, betel chewing, cannabis, and other substances. The source of knowledge regarding self-harm methods was also examined, categorized as family, friends, community, or literature.

The data was analyzed using IBM SPSS Statistics for Windows, Version 21.0 (Released 2012; IBM Corp., Armonk, New York, United States). Descriptive statistics, such as means and proportions, were used to summarize the data, while categorical variables were presented as percentages. This statistical approach facilitated a comprehensive understanding of the key factors associated with deliberate self-poisoning among the study population. While this study provides valuable insights, it has some limitations. Being conducted at a single hospital limits the generalizability of the findings, as the results may not fully represent patterns in other regions.

## Results

A total of 101 patients met the inclusion criteria for admission due to deliberate self-poisoning. The age distribution revealed that the largest group (54.5%) comprised individuals aged 18-29 years, followed by those aged 14-18 years (22.8%). Patients older than 60 years accounted for only 1% of the total cases, indicating a significant decline in self-poisoning with age. Regarding gender, 54.5% of the patients were women, while 45.5% were men.

Regarding educational background, the majority of patients (52) had completed education up to ordinary level exam (51.5%), while 26 had only primary education (25.7%). A small proportion, four (3.9%), had tertiary level education, and three (2.9%) were illiterate.

For employment status, 43 (42.5%) of the patients were employed, 32 (31.6%) were unemployed, and 26 (25.7%) were students or dependents. Most patients, 68 (67.3%), reported an income level of less than 25,000 LKR or were financially dependent on others. Age and occupation were significantly associated with the causes of deliberate self-poisoning (p<0.05).

Concerning substance abuse, it was prevalent among 34 (33.7%) of the patients, with 24 (23.7%) reporting alcohol use, 11 (10.8%) smoking, 15 (14.8%) chewing betel, and three (2.9%) using cannabis. Additionally, 15 (14.8%) of the patients abused multiple substances, and 20 (19.8%) were actively abusing substances, particularly alcohol, at the time of the self-poisoning incident. Notably, all patients with active substance abuse were men.

The types of poisons used for DSH widely varied. However, yellow oleander (37.6%) accounted for the majority of cases (38). Other substances included paracetamol, 19 (18.8%); drugs other than paracetamol, 11 (10.8%); organophosphates, six (5.9%); pesticides other than organophosphates, six (5.9%); and cerebral mango, five (4.9%). A small percentage of patients used kerosene, four (3.9%), or multiple drugs, one (0.9%), as outlined in Table 1.

**Table 1:** Type of poisons used for deliberate self-harm (n=101)

Type of poison	Number of patients (% of total cases)
Yellow oleander	38 (37.6%)
Cerebral mango	5 (4.9%)
Organophosphate	6 (5.9%)
Pesticides other than organophosphate	6 (5.9%)
Run Rat	2 (1.9%)
Paracetamol	19 (18.8%)
Drugs other than paracetamol	11 (10.8%)
Multiple drugs	1 (0.9%)
Kerosene	4 (3.9%)
Others	9 (8.9%)

Patients learned about self-poisoning methods from various sources. Fifty-three (52.5%) acquired this knowledge through word of mouth in the community. Friends were the source for 34 (33.7%), while six (5.9%) learned from family members and eight (7.9%) from literature, including newspapers, social media, and articles. Additionally, 12 (11.8%) of the patients had a history of previous suicide attempts, and three (2.9%) had a prior psychiatric disorder.

The immediate stressors that triggered DSH were identified (Table 2). The data highlights that arguments with parents were the primary stressor, with 29 (28.7%) of patients citing these conflicts as a major factor contributing to their self-harming behavior.

**Table 2:** Immediate stressors that trigger deliberate self-harm (n=101)

Immediate stressors	Number of patients (% of total cases)
Argument with spouse	19 (18.8%)
Argument with children	2 (1.9%)
Argument with parents	29 (28.7%)
Argument with other people	13 (12.9%)
Exam stress	2 (1.9%)
Argument with physical assault by husband	2 (1.9%)
Conflict regarding romantic relationship	14 (13.8%)
Severe financial difficulties	8 (7.9%)
Unbearable pain	6 (5.9%)
Bereavement of a close family member	2 (1.9%)
Others and undisclosed the reason	4 (3.9%)

Regarding the time between poisoning and hospitalization, 76 (75.2%) of patients were admitted to the hospital within six hours of poisoning, 11 (10.8%) between six and 12 hours, 10 (9.9%) between 12 and 24 hours, two (1.9%) between 24 and 48 hours, and two (1.9%) more than 48 hours after poisoning.

The study also found that 50 (50%) of patients reported experiencing immediate stressors directly related to the COVID-19 pandemic. These stressors included financial loss due to reduced income, job loss, and severe mental distress.

Finally, patient outcomes showed that 100 (99%) of patients were successfully discharged after treatment, while one (1%) case resulted in death.

## Discussion

The findings of this study are consistent with existing literature on the demographic, socioeconomic, and psychological factors contributing to DSH by poisoning. The study highlights a significant prevalence of self-harm among younger age groups, particularly those aged 18-29. This observation aligns with previous research, which investigated the epidemiology of deliberate self-poisoning in Sri Lanka and other regional countries [10-13]. These findings underscore that younger individuals remain the primary demographic at risk for this behavior. 

Further, understanding the gender distribution of patients who engage in DSH by poisoning is essential for developing targeted interventions. The study observed a slightly higher percentage of women (54.5%) engaging in poisoning-related self-harm compared to men. Previous research conducted in other regional areas in the country also reported that women were more likely to engage in deliberate self-poisoning, particularly using pesticides and oleander [14-16]. The findings suggest that gender-specific mental health interventions are needed to address the unique challenges faced by women, thereby improving clinical outcomes and ensuring efficient resource allocation.

Educational attainment plays a significant role in the likelihood of self-harm, as evidenced by this study's finding that 51.5% of patients had education levels up to ordinary level exam with limited educational level qualifications. This result is supported by existing studies, which found a correlation between lower educational levels and increased risk of self-harm [17,18]. Limited education may contribute to self-harm due to reduced access to coping resources, lower socioeconomic status, and limited awareness of mental health support services [19]. This suggests that lower educational attainment is a significant factor in the prevalence of DSH by poisoning. Understanding these educational demographics can help in tailoring preventive measures and support systems more effectively to address and reduce the incidence of self-harm in this population.

The study's findings, which revealed that 42% of the patients were employed, challenge the assumption that employment alone provides sufficient stability to prevent self-harm. While unemployment is often associated with higher rates of self-harm, the fact that a significant proportion of employed individuals also engage in self-harm underscores the complexity of this issue. The result shows that while employment may provide some stability, it does not necessarily prevent self-harm behaviors. The relatively high percentage of employed individuals engaging in DSH highlights the need for mental health support and intervention across all employment statuses and in the workplace.

The analysis indicates that patients with lower income levels are more prone to engaging in DSH by poisoning, while higher income levels correlate with a reduced incidence of such behaviors. This trend aligns with findings from previous research conducted in various regions in Sri Lanka [20]. This economic disparity suggests that financial status can significantly impact self-harm tendencies. Recognizing these income-related factors can aid in developing targeted intervention strategies that address the economic challenges faced by specific groups.

The data reveals significant insights into the substances most frequently involved in DSH by poisoning in the study population. The predominance of yellow oleander as the most commonly used poison, accounting for 37.6% of incidents, highlights the ease of access to this toxic plant in Sri Lanka and other tropical regions. Previous research conducted in rural Sri Lanka identified yellow oleander seeds as the primary substance used in DSH [10]. Nearly two decades later, yellow oleander remains a leading cause of self-poisoning, highlighting the persistent challenge it poses. This continued prevalence suggests the need for a thorough review of existing intervention mechanisms and the identification of any gaps in current strategies. Strengthening and refining these efforts is crucial to reducing the incidence of self-harm associated with yellow oleander.

Following yellow oleander, paracetamol emerges as the second most common substance involved in self-poisoning cases, responsible for 18.8% of incidents. Paracetamol is a widely available over-the-counter medication, and its frequent use in self-harm may be attributed to its accessibility and the public's awareness of its potential lethality in large doses. This finding aligns with global trends, where paracetamol is a common agent in self-poisoning due to its easy availability [21]. Drugs other than paracetamol make up 11.9% of cases, indicating that pharmaceuticals are a significant contributor to self-harm incidents. This points to the necessity for stricter regulation and control of access to medications, particularly in households where vulnerable individuals may be at risk of self-harm.

The data reveals critical insights into the immediate stressors that trigger DSH, shedding light on the complex interplay of familial, relational, and personal challenges faced by individuals. The most prominent stressor identified is conflict with parents, reported by nearly 29% of patients, indicating the significant impact of parent-child relationships on mental health. This suggests that familial discord, particularly with parents, plays a crucial role in pushing individuals towards self-harm, potentially due to unmet expectations, communication breakdowns, or generational conflicts.

Marital disputes and conflicts in romantic relationships also emerge as key stressors. Overall, the data suggests that addressing the underlying causes of these stressors through targeted interventions, such as family counseling, relationship support, financial assistance, and pain management, could be crucial in preventing self-harm. By understanding the specific triggers that lead individuals to self-harm, mental health professionals can develop more effective, tailored approaches to support those at risk.

Substance abuse, particularly alcohol and smoking, was identified as a significant risk factor in this study, with 33.7% of patients having a history of substance use. The use of substances can impair judgment, reduce inhibitions, and increase impulsivity, making individuals more likely to engage in self-harm. The high prevalence of substance abuse among self-harm patients underscores the need for integrated treatment approaches that address both mental health and substance use disorders.

The study's exploration of sources of knowledge about self-harm methods revealed that community engagement and social circles play a significant role in disseminating information about self-harm. Public health strategies should focus on disrupting these social networks and providing positive role models to reduce the incidence of self-harm.

To support the prevention of further DSH, patients who had been admitted for poisoning were followed up in psychiatric or mental health clinics. In terms of patient outcomes, the study revealed that the vast majority of individuals who were hospitalized following poisoning incidents around 99% were successfully treated and discharged from the hospital. This high discharge rate indicates the effectiveness of timely medical intervention and the hospital's capability to manage poisoning cases. However, despite these positive outcomes, the study also recorded a 1% mortality rate among the patients. This small but significant percentage highlights the critical importance of rapid response and effective treatment in poisoning cases, as delayed hospitalization or the severity of the poisoning can still lead to fatal outcomes. These findings are consistent with global benchmarks for poisoning treatment outcomes, where similar mortality rates have been observed. The fatality could potentially have been avoidable with timely intervention and access to appropriate mental health support and medical treatment. However, certain self-harm attempts involve ingestion of highly toxic substances, which often carry a high likelihood of mortality regardless of intervention.

## Conclusions

This study reveals that younger individuals, particularly those aged 18-29, women, and people with lower education and income levels, are more vulnerable to deliberate self-poisoning. Substance abuse, especially alcohol, and familial conflicts, particularly with parents and spouses, were major triggers for self-harm. The impact of the COVID-19 pandemic further exacerbated these stressors, with half of the patients citing pandemic-related financial loss, job loss, and mental distress as key factors in their decision to self-harm. These findings highlight the need for targeted mental health interventions focusing on at-risk groups to prevent DSH.
